# Associations between health-related quality of life and demographics and health risks. Results from Rhode Island's 2002 behavioral risk factor survey

**DOI:** 10.1186/1477-7525-4-14

**Published:** 2006-03-03

**Authors:** Yongwen Jiang, Jana Earl Hesser

**Affiliations:** 1 Rhode Island Department of Health, 3 Capitol Hill, Providence, RI 02908

## Abstract

**Background:**

Health-Related Quality of Life (HRQOL) has received much attention in recent years. HRQOL indicators have been used to track population trends, identify health disparities, and monitor progress in achieving national health objectives for 2010. Prior studies have examined health risks and HRQOL at the national level as well as at the state level. This paper examines multiple indicators of HRQOL by demographic characteristics and selected health behaviors for Rhode Island adults.

**Methods:**

Data from Rhode Island's 2002 Behavioral Risk Factor Surveillance System (BRFSS), a random digit dialled telephone survey, were used for this study. The state wide sample contained a total of 3,843 respondents ages 18 and older. Multiple Imputation (MI) was applied to handle missing data, and data were modelled for each of 10 HRQOL indicators using multivariable logistic regression.

**Results:**

By examining HRQOL through a multivariable approach we identified the strongest predictors for multiple indicators of poor HRQOL as well as predictors for specific indicators of poor HRQOL. Predictors for multiple indicators of poor HRQOL were: disability, inability to work, unemployment, lower income, lack of exercise, asthma, and smoking (specifically associated with poor mental health).

**Conclusion:**

Using multiple measures of HRQOL can help to assess the burden of poor health in a population, identify subgroups with unmet HRQOL needs, inform the development of targeted interventions, and monitor changes in a population's HRQOL over time. Use of these HRQOL measures in longitudinal and intervention studies is needed to increase our understanding of the causal relationships between demographics, health risk behaviors, and HRQOL.

## Background

One of the two overarching goals of Healthy People 2010 is to increase the quality and years of healthy life for all people in the United States [1]. It is estimated that 41 million American adults 18 and older experience physical or mental impairments that affect their quality of life [2]. In public health and in medicine, the concept of health-related quality of life (HRQOL) refers to "those aspects of general subjective quality of life affecting a person's health or health perceptions," [3] or to a person's "perceived physical and mental health" [4].

In the early 1990's, responding to concerns about the aging of the population, the burden of chronic disease, the epidemics of obesity and diabetes mellitus, environmental health threats, the persistence of health disparities and the performance and cost of health care services, the Centers for Disease Control and Prevention (CDC) developed and validated a set of four "core" survey questions to track HRQOL in states and communities [3,5]. Tracking HRQOL can identify subgroups with poor physical or mental health, can help guide policies or interventions to improve population health, and can monitor progress towards national health goals [4,6-10]. These HRQOL indicators assess dysfunction and disability, providing broad measures of population health not reflected in the standard measures of morbidity and mortality.

Since January 1993, the four "core" HRQOL questions [10,11] have been included on CDC's Behavioral Risk Factor Surveillance System (BRFSS) questionnaire to track adults' perceptions of their general health status, physical and mental health, and activity limitations related to physical or mental health. The Behavioral Risk Factor Surveillance System is an ongoing, state-based, annual random-digit-dialed telephone survey of the non-institutionalized civilian population ages 18 and older. It has been sponsored since 1984 by the Centers for Disease Control and Prevention (CDC), which provides funding, methodological specifications, and technical assistance to participating states. The BRFSS is conducted currently in all 50 states and in four U.S. territories [8]. In addition to providing demographic information, respondents answer a variety of questions about health status, health conditions, health risk behaviors, health insurance coverage, and access to care. The BRFSS monitors the prevalence of behavioral risks for the leading causes of disease and death among adults in the United States [8]. A detailed description of BRFSS methods, including survey design, and random sampling and weighting procedures, is available elsewhere [12].

In response to growing interest in HRQOL, an expanded set of HRQOL questions was developed for use on the BRFSS which have been available since 1995. These questions measure multiple dimensions of HRQOL including "specific types of activity limitation [including Activities of Daily Living] and common physical and emotional symptoms" [11]. Rhode Island has included the "core" and expanded HRQOL questions on its annual BRFSS since 1997. In 2000 Rhode Island adopted HRQOL as an overarching goal for its Healthy People 2010 initiative.

Prior studies have examined health risks and HRQOL at the national level [4], and a few state-level studies have been published [6,13,14]. In this paper, 2002 BRFSS data for Rhode Island are analyzed to assess the relationship between demographics, health risks, and health conditions as independent variables and each of ten HRQOL indicators.

## Methods

### Data source

Data from Rhode Island's 2002 BRFSS were used for this analysis. Rhode Island has participated in the BRFSS since 1984. A professional survey research organization conducts the annual survey under contract to the Rhode Island Department of Health's Center for Health Data and Analysis. Using a disproportionate stratified random sampling design, approximately 320 random – digit dialed telephone interviews were completed with Rhode Island adults each month during 2002, for a total of 3,843 during the calendar year. Data were weighted to adjust for the probability of respondent selection and post-stratified to reflect the age and sex distribution of Rhode Island's estimated adult population during 2002. Technical details of RI's 2002 BRFSS survey are available from the Rhode Island Department of Health Center for Health Data and Analysis [15]. The response rate in 2002, as defined by the Council of American Survey Research Organizations (CASRO) was 51%. Rhode Island BRFSS data is available upon request from the Center for Health Data and Analysis, Rhode Island Department of Health.

### Variables

Nine of the HRQOL questions (Appendix A; See Additional File 1) asked as part of the 2002 Rhode Island BRFSS were used as dependent variables:

• self-rated general health status

• self-reported number of healthy/unhealthy days in the past 30 days for -

• physical health

• mental health

• physical or mental health related activity limitation

• pain related activity limitation

• sad, blue or depressed

• worried, tense or anxious

• lack of sleep

• lack of energy [16]

In addition, in 2002 respondents answered a set of questions added to Rhode Island's BRFSS which were derived from the 1997 National Household Survey on Drug Abuse (NHSDA). These questions were used with an algorithm specified by NHSDA to generate a single variable indicating the occurrence in the prior year of a major depressive episode (MDE) [17]. MDE was included in the analysis as a tenth HRQOL indicator.

The responses to the general self-rated health status question were dichotomized into "poor" (poor or fair health) or "good" (good, very good, or excellent health) health.

The items measured in days were dichotomized at a cut-off value of 14 or more days of poor health in the past month vs. less than 14 days [16]. The 14-day minimum period was selected because clinicians and clinical researchers often use this period as a marker for clinical depression and anxiety disorders, and longer symptom durations are associated with a higher level of activity limitation [4,18]. In addition, most of the publications we reviewed based on the BRFSS HRQOL indicators use the cut-off of 14 or more days vs. 13 or fewer days [2,7,16,19-25]. We chose to adopt this precedent for comparability. MDE was dichotomized as "occurred in the past 12 months" vs. "did not occur in the past 12 months".

Twelve independent variables (Appendix B; See Additional File 2) selected for inclusion in the analysis were:

• five standard demographic measures -

• age

• sex

• race/Hispanic ethnicity

• income

• employment

• four health conditions -

• asthma

• diabetes

• obesity

• physical disability

• three health risk behaviors -

• smoking

• chronic alcohol use

• no leisure physical activity

Selection of these independent variables was based on results of other studies which have found relationships between a specific HRQOL indicator and various independent variables [20,26], or which have examined multiple HRQOL indicators in relation to specific health conditions [21-23,27-29], health risks [7,9,24], or demographics [19,30]. In addition, preliminary modelling identified these as important variables to retain while a number of others were eliminated. For analysis, some independent variables were dichotomized (sex, current smoking, alcohol use, physical activity, asthma, diabetes, obesity, disability); others with multiple or continuous response categories (age, race/Hispanic ethnicity, income, and employment status) were reduced to the smallest number of meaningful categories yielding at least 5 cases or more in any table cell. Reference groups for the multivariable logistic regression (MLR) were chosen as those having the lowest risk for poor/fair general health (and usually lowest risk for the other HRQOL variables); they also had large sample sizes.

### Analysis

The goal of the analysis was to assess the effect of each independent variable on each of the HRQOL indicators by using a multivariable logistic regression (MLR) modelling procedure.

SAS 9.1 [31] was used for all analyses because it can adjust for the BRFSS' complex sampling design. Using the SAS SURVEYFREQ procedure, we first obtained prevalence estimates and standard errors of estimates for each indicator of poor HRQOL, in relation to each of the 12 independent variables. The χ^2 ^test was used to identify statistically significant relationships (p (two-sided) ≤ 0.05).

In the next step, using logistic regression, crude (unadjusted) odds ratios (CORs) and χ^2 ^tests of significance were calculated for each of the 12 independent variables in relation to each of the poor HRQOL indicators. CORs make it possible to evaluate the strength of the relationship between independent variable categories and dependent variables but does not account for confounding effects between variables. Using SAS PROC SURVEYLOGISTIC, a multivariable logistic regression (MLR) procedure was used to calculate adjusted odds ratios (AORs) and 95% confidence intervals (CIs) to assess the effect of each of the 12 independent variables for each of the poor HRQOL indicators. The MLR procedure adjusts for possible confounding effects between independent variables and helps identify those which are the best predictors for each of the poor HRQOL indicators. No two-way interactions between the independent variables were found to be significant. All statistical inferences were based on a significance level of p (two-sided) ≤ 0.05.

When modelling with MLR using SAS PROC SURVEYLOGISTIC, if any record contains a missing value for any variable in the analysis, that record along with all of its valid data, is eliminated from the entire analysis [32]. In our study, depending on the specific HRQOL model, 24.8% – 27.3% of the 3,843 records (953 – 1048 records) were excluded from the initial MLR calculations because of missing data for any one of the dependent or independent variables. This "available-cases" analysis could yield biased estimates if there were systematic differences between those included in the analysis and those excluded because of missing data [33]. In addition, the ten "available-cases" subsets for the ten logistic regression models would be different, making comparison of results of the ten models difficult. Consequently, in order to retain all valid data, maintain maximal sample size for the MLR, and make the datasets the same for each of the 10 HRQOL models, we simulated missing data for all variables using multiple imputation (MI). MI is a procedure recommended even when 20% to 30% of data are missing [32].

MI has been extensively applied to handle missing data in survey sampling [34,35]. The method of MI we used is a Markov Chain Monte Carlo technique in which the missing values ("m") are replaced by m>1 simulated versions of the data, where "m" is typically small (say, 3–10) [36]. In our study, 6 complete datasets were created by replacing missing values with simulated values. When doing multiple imputations, it is appropriate to include as many variables as possible to impute values for missing demographic and risk factor variables [37]. The imputation model, using SAS PROC MI, included the variables of age, sex, race, marital status, education, employment status, health insurance, smoking, drinking, physical activity/exercise, asthma, diabetes, obesity, disability, square-root-transformed income, and the 10 HRQOL variables.

Before we did multiple imputations, we checked the distribution of every variable. The original income variable has eight categories. When treated as a continuous variable we found it was not normally distributed. Consequently, the income variable was "square-root-transformed" for the MI. After MI, we regrouped the income variable into three categories for the MLR.

A basic assumption of MI is that missing data are missing at random [34]. We compared the MLR results for each model "before MI" and "after MI" to assess the impact of simulating missing data on AORs. The AORs did not change dramatically indicating that missing data were missing at random.

After MI, each of the simulated complete datasets was analyzed by standard methods, and the results combined through the process of multiple imputation inference to obtain parameter estimates (AORs) and CIs that incorporate missing-data uncertainty (SAS PROC MIANALYZE) [36]. The same set of covariates was used for adjustment in each multivariable logistic regression model.

## Results

### Table 1 (attached as Additional File 3)

Table 1 presents prevalence estimates and 95% confidence intervals (CIs) for HRQOL indicators by demographic, health condition, and health risk variables. Overall, 13.7% had fair or poor general health. Results for the 8 indicators based on the criterion of 14 or more days of poor health in the past month (hereafter referred to as recent frequent [RF] poor health) were as follows: 4.8% had RF activity limitations due to a physical or mental health problem; 9.7% had RF poor physical health; 7.2% had RF pain related activity limitations; 27.9% reported RF lack of energy; 9.5% had RF poor mental health; 8.0% were RF sad, blue or depressed; 12.6% were RF worried, tense or anxious; and 23.9% reported RF inadequate sleep or rest. A major depressive episode in the past 12 months was experienced by 7.6%.

Each of the independent variables was significantly associated with 4 or more indicators of poor HRQOL. The following are general observations about prevalence patterns identified across HRQOL indicators for each of the independent variables.

### Age

The prevalence of three of the indicators for poor physical health (fair/poor general health, RF physically unhealthy days, and RF pain related activity limitation) increased with increasing age, while the prevalence of indicators for RF poor mental health decreased with increasing age. All differences were statistically significant. There were no significant age related differences for RF physical or mental health related activity limitations and RF lack of energy.

### Sex

Women reported a higher prevalence than men for all of the HRQOL indicators. All differences were statistically significant except for "RF pain related activity limitations" and "RF lack of rest/sleep".

### Race/Hispanic ethnicity

It was necessary to group all non-white, non-Hispanic respondents into a single "other" category due to small sample sizes. There were significant differences by race/Hispanic ethnicity for 5 of the 10 indicators. "Hispanics" had the highest percent with "poor/fair" general health, but did not have the highest percent for other indicators of RF poor HRQOL. The "Other" group had the highest percentages for "RF lack of energy", "RF lack of rest/sleep", for "RF sad/blue depressed" and for MDE and these were significant differences.

### Income

The prevalence of all poor HRQOL indicators increased with decreasing levels of annual household income. All differences were statistically significant with the exception of "RF lack of rest/sleep".

### Employment

Statistically significant differences occurred for all poor HRQOL indicators in relation to employment status. Respondents who were unable to work had higher rates than other employment categories for all indicators. Respondents who were unemployed also had higher rates than others for most of the poor HRQOL indicators although they were very similar to retired persons for poor general health, RF physically unhealthy days, and RF pain related activity limitations.

### Tobacco use

Current smokers had significantly higher rates than non-smokers for all poor HRQOL indicators except for general health status.

### Physical inactivity

Adults who were physically inactive had significantly higher rates for all poor HRQOL indicators than adults who reported engaging in leisure time physical activity.

### Chronic alcohol use

Chronic drinkers were significantly less likely to report poor general health or poor physical health than those who were not chronic drinkers, but significantly more likely to report "RF worried tense or anxious" or "RF lack of rest or sleep".

### Diabetes and asthma

People who had been told by a physician that they had diabetes or asthma had significantly higher percentages for all indicators of poor HRQOL than persons without these conditions, with the exception that diabetics and non-diabetes were equally likely to report RF lack of rest or sleep.

### Obesity and disability

Obese persons and disabled persons had higher percentages for all poor HRQOL indicators than non-obese or non-disabled persons, and all differences were statistically significant.

### Table 2 (attached as Additional File 4)

Table 2 presents the crude (unadjusted) odds ratios (CORs), and 95% confidence intervals (CIs) for demographic, health condition, and health risk variables regressed on HRQOL indicators. The CORs make it possible to evaluate the strength of the relationship between independent variable categories and dependent variables without adjusting for confounding effects. The highest CORs occurred among those unable to work for all but two of the HRQOL indicators. Those with disabilities had the next highest CORs for all but three indicators of poor mental health. The CORs for those indicators were comparable or only slightly less than those for the unemployed.

### Table 3 (attached as Additional File 5)

Table 3 presents the adjusted odds ratios (AORs), and 95% confidence intervals (CIs), for demographic, health condition, and health risk variables regressed on HRQOL indicators using multivariable logistic regression (MLR) after multiple imputation. The same set of co-variables was used for adjustment in each MLR model.

In comparing the AORs with the CORs, the number of variables significantly related to the HRQOL variables decreased after adjustment, and almost all of the significant AORs were lower than the CORs, some substantially, indicating the presence of confounding effects (compare Table 2 and 3) (attached as Additional File 4 and 5).

The AORs in most instances were greater than 1, indicating a higher probability of poor HRQOL when compared with the reference group. However, in a few instances the AORs were less than 1 indicating decreased risk compared with the reference group.

Significantly increased AORs occurred for five or more of the poor HRQOL indicators for seven of the independent variables: have disability, unable to work, no leisure physical activity, have asthma, household income <$25 K, current smoker and unemployed. Age 65+ had decreased AORs associated with the indicators of poor mental health.

Being physically disabled had significant increased AORs for all ten of the HRQOL indicators and had the highest AORs associated with the variables related to physical health, e.g. general health status (4.5), RF activity limitation (10.6), RF poor physical health (5.3), and RF pain related activity limitation (7.8). It was also the strongest predictor for four of the six indicators of poor mental health – RF lack of energy (3.4), RF worried/tense/anxious (2.6), RF lack of rest/sleep (2.8), and major depressive episode (3.3) and is significantly elevated for RF poor mental health (2.2) and for RF sad/blue/depressed (2.5).

After the MRL, the AORs were much lower than the CORs for "unable to work" indicating that other independent variables (e.g. disability) are important confounders for being unable to work. Nonetheless, being unable to work was the second strongest predictor of both poor physical and mental HRQOL, with AORs between 1.9 and 5.0. It had the highest AORs of any variable for RF poor mental health (2.7) and for RF sad/blue/depressed (3.7). Being unable to work was not a significant predictor for RF lack of rest/sleep (1.1).

"No leisure time physical activity" was a significant predictor (with AORs between 1.5 and 1.8) for eight of the ten poor HRQOL indicators, the exceptions being RF lack of rest/sleep and MDE.

Current asthma was a significant predictor for six of 10 indicators including poor general health status (1.9), RF activity limitation (1.8), poor physical health (2.4), RF lack of energy (1.5), RF worried/tense/anxious (1.6), and MDE (2.2).

Being unemployed significantly increases the odds for poor general health (2.4), RF activity limitations (2.2) and for four of the mental health indicators – poor mental health (1.8) RF sad/blue/depressed (1.9), RF worried/tense/anxious (2.3) and MDE (1.8).

Persons with household incomes less than $50,000 had significantly greater odds (AORs 1.7 – 2.9) than higher income persons for poor/fair general health, RF sad/blue/depressed, and for RF worried/tense/anxious. Having household income less than $25,000 was a significant predictor as well for RF lack of energy (1.7), and RF poor mental health (1.8).

Hispanic ethnicity was a strong predictor for poor/fair general health (4.1) but this finding seemed anomalous when compared with the significantly decreased risk for Hispanics for RF lack of energy (0.7) and with the non-significant results for all other indicators.

Diabetics experience double the odds (2.1) of poor/fair general health compared with non-diabetics, and have increased odds of having RF pain related activity limitation (1.7) and for RF sad/blue/depressed (1.7).

Current smoking was a predictor for five mental health indicators: RF poor mental health (1.5), RF sad/blue/depressed (1.8), RF worried/tense/anxious (1.7), RF lack of rest/sleep (1.4) and MDE (1.9) but not for RF lack of energy nor for any of the poor physical health indicators.

Females had significantly increased odds compared with males for four mental health indicators: RF poor mental health (1.4), RF worried/ tense/ anxious (1.4), RF lack of rest/sleep (1.3) and MDE (1.7) but not for RF sad /blue /depressed.

The AORs for poor general health increased with each of the two older age groups – from 2.1 for those ages 45 – 64 to 2.5 for those ages 65 and older. However, when other variables are controlled, the oldest age group was not at increased risk for other indicators of poor HRQOL. In fact, this age group had decreased odds (0.5) for RF activity limitations when compared with younger adults as well as significantly decreased odds compared with younger adults for each of the indicators for poor mental health. The AORs for RF lack of rest/sleep was less than one for both groups of older adults (0.6 and 0.3), in relation to the reference group aged 18 – 44. Those 45–64 had significantly greater odds (1.5) than the reference group for RF pain related activity limitations.

Obesity did not have significant elevated AORs for any of the HRQOL indicators other than for RF poor/fair general health (1.4) and RF lack of energy (1.3). Chronic drinking was a significant predictor only for RF worried/tense/anxious days (2.0).

## Discussion

These results from simultaneously modelling 10 HRQOL indicators and 12 independent variables are consistent with and extend previous studies [2,26] documenting the relationship between a variety of measures of poor HRQOL and certain demographics, risk behaviors, and health conditions. They also identify new relationships, suggest avenues for further investigation, and suggest populations to target for health interventions.

By examining HRQOL through a multivariable approach we identified the strongest predictors for multiple indicators of poor HRQOL. These results may enable targeting of specific populations at high risk for poor HRQOL. These high risk populations include: the disabled (defined by the screening criteria employed by the BRFSS), people who are unable to work or who are unemployed, people with lower incomes, people who do not exercise, asthmatics, and smokers (specifically at risk for poor mental health).

Physical disability had significant high AORs across all ten indicators for poor HRQOL and was the strongest predictor overall of poor HRQOL. While the high AORs associated with poor physical health and limitations are not surprising, disability also had significant increased AORs for the poor mental HRQOL indicators. Our results, which support others [11], indicate that not only are disabled persons physically limited and in poor physical health, but they also are especially vulnerable to pain, depression, anxiety, sleeplessness, and lack of vitality. A substantial segment (~20%) of RI's population has disabilities [38]. Secondary prevention strategies could be designed to improve access to treatment for pain, depression and other mental health conditions for persons with disabilities.

Being unable to work was the second strongest overall predictor of poor HRQOL. Persons who are unable to work have poor physical health, even when controlling for physical disability, and being unable to work was the strongest predictor for two of the poor mental health indicators. Being unemployed had increased AORs for 6 of the indicators, 4 of them for mental health, but it is not as strong a predictor of poor HRQOL as being unable to work. However, the strong relationship between poor HRQOL and being unable to work or unemployed may indicate that work is an important component of good HRQOL. Work increases access to health care by providing health insurance, may offer social networks, assures income, and may give meaning to daily life. On the other hand, poor HRQOL may lead to unemployment or inability to work. Causal inferences cannot be drawn from results of cross-sectional data.

In this study, the strong association between physical inactivity and poor HRQOL is striking. Lack of physical activity was a significant predictor for 8 indicators of poor HRQOL. The benefits of physical activity for health are well documented. Regular physical activity is associated with a decreased risk of cardiovascular disease and all-cause mortality and has favorable effects on blood pressure, lipid and lipoprotein profiles, weight control and body fat distribution, as well as on mental health [7,39].

Current asthma increased the odds of poor HRQOL for six indicators of both physical and mental HRQOL; diabetes increased the odds of poor HRQOL for 3 indicators. Asthma and diabetes have been found to have an effect on HRQOL in previous research [21], and depression is common in people with chronic conditions [11]. Secondary prevention strategies could be designed to enhance HRQOL for persons with these chronic conditions.

Hispanic ethnicity was a strong predictor for poor/fair general health, but had non-significant results for other indicators of poor HRQOL. These results seem contradictory and suggest that Hispanics may understand the general health status question differently from other respondents, or they may use different criteria from other groups in assessing their general health [40].

Annual household income of $25,000 or less was a predictor for 5 indicators, with 4 of them being indicators of poor mental health rather than for indicators of RF poor physical health or activity limitations. These results are consistent with other studies that have documented strong associations between lower socioeconomic status and poorer health outcomes but the lack of association with indicators of poor physical health or limitations is of interest [11].

Current smoking, and being female were significant predictors only for indicators of poor mental health. Other studies have shown that women experience a higher prevalence of mental health impairment than men [2,11] and smoking rates are higher among persons with mental illness [41].

The older adults (65+) represented in our sample are mentally healthier than younger adults (ages 18 – 64), and little different in physical health. These surprising results likely represent bias in the sampling of older adults. Older adults who are healthy and residing in their own homes are eligible to participate in a phone survey while those who are less healthy may be less able to participate in a phone survey, or may not be living independently. Residents in institutional or group settings, such as nursing homes, are excluded from the sample of adults reached by the BRFSS. However, it is also possible that older adults are less likely to recognize or be willing to report psychological problems. It is also possible that controlling for variables that are strong predictors for poor HRQOL reduces the importance of age as an independent variable related to poor HRQOL. For example, disability, that becomes more prevalent with increasing with age, is a significant factor contributing to poor HRQOL.

In our study, obesity was not a significant predictor for poor HRQOL when other variables (e.g. disability, low income) were controlled. This was unexpected because other studies indicate that obesity is associated with increased health risks that can impair physical health status and impose limitations on daily activities [23], and also has an effect on mental health [28,29]. Other researchers have suggested that poor emotional health among the obese might be due to a higher prevalence of co-morbidities rather than to the obesity itself [29] and our results support this position. Obesity is associated with diabetes mellitus, hypertension, coronary heart disease, respiratory disease, osteoarthritis, and mobility impairment, and such co-morbidities could be major factors impacting HRQOL [29].

### Limitations

Our study has certain limitations. First, because BRFSS is a telephone survey, it excludes the homeless, residents of institutionalized settings such as nursing homes, and people without a telephone who tend to be primarily of lower SES. Such people may have a higher prevalence of health impairments or activity limitation and their exclusion from our sample may introduce selection bias and under estimation of the prevalence of poor HRQOL and the strength of associations between poor HRQOL and the independent variables. Second, people with decreased physical and mental capacity might not have been able to complete the survey, which could lead to under representation in the sample of people with poor HRQOL. Although BRFSS weighting procedures are assumed to control for non-coverage and non-response bias it may be inadequate to control for certain types of selection bias. Third, because the data are self-reported, they might be subject to recall bias and under or misreporting of health behaviors, health conditions, and HRQOL. For example, underreporting of weights and over-reporting of height is particularly characteristic among overweight and obese subjects, especially among women and the elderly [22,29], resulting in misclassification of individuals and underestimates of overweight and obesity. Fourth, BRFSS data, which are cross-sectional, does not allow us to draw inferences about causal relationships between poor HRQOL and other respondent characteristics.

## Conclusion

Additional studies focusing on distinct populations at increased risk for poor HRQOL (e.g. disabled, those unable to work, smokers) are needed to understand causal relationships and such understanding can help in the design of strategies for primary prevention. Prior studies show that those with physical and mental impairments are more likely to engage in high-risk behaviors or to be at risk for adverse health conditions [2,22,26,42] but understanding what leads to these correlations in the data is more difficult. Longitudinal studies and qualitative studies would be of considerable value.

These results can also help in designing secondary prevention interventions that will have maximum impact for the HRQOL of Rhode Island's population. Limited resources can be utilized most effectively by identifying and focusing interventions on those populations most vulnerable to poor HRQOL. For example, our results indicate the need to provide improved access to treatment for pain and depression for people who have asthma, or people who have disabilities. Likewise, the significant association between poor mental HRQOL and smoking suggests that reducing tobacco rates in the state requires that tobacco cessation programs address behaviors and needs other than just smoking behaviors, or that mental health services and smoking cessation services need to work closely together for cessation efforts to be successful. If we are to meet Healthy Rhode Island 2010 goals of increasing quality and years of healthy life, and eliminating health disparities, we need to understand and target those populations most at risk for poor HRQOL [1,10].

## Abbreviations

AORs: Adjusted Odds Ratios

BRFSS: Behavioral Risk Factor Surveillance System

CDC: Centers for Disease Control and Prevention

CIs: Confidence Intervals

CORs: Crude Odds Ratios

HRQOL: Health-Related Quality of Life

MI: Multiple Imputation

MLR: Multivariable Logistic Regression

RF: Recent Frequent

## Competing interests

The author(s) declare that they have no competing interests.

## Authors' contributions

YJ contributed to preparation of the database, conducted the literature review, collaborated on analytic decisions and data interpretation, performed the statistical analyses, prepared data tables, and drafted the manuscript. JEH manages the RI BRFSS, collaborated on analytic decisions and data interpretation, and revised and edited the manuscript. Both authors have read and approved the final version of the manuscript.

## Supplementary Material

Additional File 1 Appendix A Click here for file

Additional File 2 Appendix B Click here for file

Additional File 3 Table 1. Percentage of HRQOL indicators for selected demographic characteristics and risk factors^†^, Rhode Island adults, 2002. Click here for file

Additional File 4 Table 2. Demographic characteristics and risk factors regressed on HRQOL indicators (Crude Odds Ratios) ^†^, Rhode Island Adults, 2002 Click here for file

Additional File 5 Table 3. Demographic characteristics and risk factors regressed on HRQOL indicators, Rhode Island Adults, 2002. (Adjusted Odds Ratios after multiple imputation) ^†^ Click here for file
