# Supplementary material for: Associations between health-related quality of life and demographics and health risks. Results from Rhode Island's 2002 behavioral risk factor survey
Source: Health Qual Life Outcomes. 2006 Mar 3;4:14. doi: 10.1186/1477-7525-4-14 (PMC1431510; doi:10.1186/1477-7525-4-14)
Supplement: Additional File 1 — Appendix A [file 1477-7525-4-14-S1.pdf]

Appendix A.

Questions from the 2002 Rhode Island BRFSS used to evaluate Health-Related Quality of Life:

1. Self-Perceived Health
   Would you say that in general your health is
   1) Excellent
   2) Very good
   3) Good
   4) Fair, or
   5) Poor?
2. Recent Physical Health
   Now thinking about your physical health, which includes physical illness and injury, for how many days during the past 30 days was your physical health not good?
   _____ days
3. Recent Mental Health
   Now thinking about your mental health, which includes stress, depression, and problems with emotions, for how many days during the past 30 days was your mental health not good?
   _____ days
4. Recent Physical or Mental Health Related Activity Limitation
   During the past 30 days for about how many days did poor physical or mental health keep you from doing your usual activities, such as self-care, work, or recreation?
   _____ days
5. Recent pain related activity limitation
   During the past 30 days, for about how many days did pain make it hard for you to do your usual activities, such as self-care, work, or recreation?
   _____ days
6. Recent sad, blue, or depressed
   During the past 30 days, for about how many days have you felt sad, blue, or depressed?
   _____ days
7. Recent worried, tense, or anxious
   During the past 30 days, for about how many days have you felt worried, tense, or anxious?
   _____ days
8. Recent not get enough rest or sleep
   During the past 30 days, for about how many days have you felt you did not get enough rest or sleep?
   _____ days
9. Recent not very healthy and full energy

During the past 30 days, for about how many days have you felt very healthy and full of energy?

_____ days

10. Major Depressive Episode is a variable derived by algorithm from responses to 14 questions from the 1997 National Household Survey on Substance Abuse. It indicates a period of two weeks or more in a row during the past 12 months when the respondent felt felt sad, blue, or depressed and also exhibited other symptoms associated with major depression such as loss of interest in most things, thoughts of death, weight loss or gain, sleep disturbance, loss of concentration, and low energy.

For Rhode Island's 2002 questionnaire go to

http://www.health.ri.gov/chic/statistics/2002RIBRFSSquest.pdf
