# Supplementary material for: Associations between health-related quality of life and demographics and health risks. Results from Rhode Island's 2002 behavioral risk factor survey
Source: Health Qual Life Outcomes. 2006 Mar 3;4:14. doi: 10.1186/1477-7525-4-14 (PMC1431510; doi:10.1186/1477-7525-4-14)
Supplement: Additional File 2 — Appendix B [file 1477-7525-4-14-S2.pdf]

Appendix B.

Items from the 2002 Rhode Island BRFSS used as independent variables:

1. Age – Three categories
   1) 18 – 44
   2) 45-64
   3) 65+

2. Sex
   1) Male
   2) Female

3. Race/Ethnicity
   1) White non-Hispanic
   2) Hispanic
   3) Other non-Hispanic

4. Household Income
   1) <$25,000
   2) $25,000 – $49,999
   3) $50,000 or more

5. Employment
   1) Unable to work
   2) Unemployed
   3) Retired
   4) Homemaker/Student
   5) Employed

6. Currently has asthma
   1) Asthma
   2) No Asthma

7. Respondent was told by a doctor that he/she has diabetes.
   1) Diabetes
   2) No Diabetes

8. Two Level Body Mass Index (BMI) categories. BMI = weight in kilograms divided by the square of height in meters (kg/m^2), and is based on self-reported height and weight.
   1) Obese (BMI>30)
   2) Not Obese (BMI $\leq$30)

9. Physical disability: Respondents limited in any way in any activity because of any physical problem, or using special equipment such as a cane, a wheelchair, a special bed, or a special telephone.
   1) Disability
   2) No disability

10. Current smoker: Respondents who had smoked at least 100 cigarettes in their lifetime and now smoke everyday or some days.
    1) Current Smoker
    2) Not Current Smoker

11. Chronic drinker: Male respondents having an average of more than 2 drinks per day; females respondents having an average of more than 1 drink per day.
    1) Chronic Drinker
    2) Not Chronic Drinker

12. No leisure time physical activity or exercise during the past 30 days other than the respondent's regular job.
    1) Leisure Time Activity
    2) No Leisure Time Activity
