# Supplementary material for: Associations between health-related quality of life and demographics and health risks. Results from Rhode Island's 2002 behavioral risk factor survey
Source: Health Qual Life Outcomes. 2006 Mar 3;4:14. doi: 10.1186/1477-7525-4-14 (PMC1431510; doi:10.1186/1477-7525-4-14)
Supplement: Additional File 3 — Table 1. Percentage of HRQOL indicators for selected demographic characteristics and risk factors†, Rhode Island adults, 2002. [file 1477-7525-4-14-S3.pdf]

Table 1. Percentage of HRQOL indicators for selected demographic characteristics and risk factors[†], Rhode Island adults, 2002.

Demographic characteristics & risk factors		Weighted Percent	Poor/fair general health	Activity limitation[‡]	Physically unhealthy[‡]	Pain related activity limitation[‡]	Lack of energy[‡]	Mentally unhealthy[‡]	Sad/blue/ depressed[‡]	Worried/tense /anxious[‡]	Lack of rest / sleep[‡]	Major depressive episode
n(weighted percent)		3843	570(13.7%)	203(4.8%)	393(9.7%)	294(7.2%)	1016(27.9%)	358(9.5%)	309(8.0%)	482(12.6%)	861(23.9%)	303(7.6%)
Age group (3793)	18-44 years	51.3	8.0(6.5-9.5)***	4.2(3.2-5.2)	6.9(5.5-8.3)***	4.8(3.5-6.1)***	27.4(24.9-30.0)	11.2(9.3-13.1)***	9.0(7.2-10.7)*	14.9(12.9-16.9)***	30.3(27.5-33.1)***	8.5(7.0-10.1)**
	45-64 years	29.6	15.5(13.3-17.8)	6.2(4.7-7.7)	10.9(9.0-12.8)	9.9(8.1-11.7)	27.1(24.1-30.1)	10.3(8.3-12.3)	8.2(6.5-9.9)	13.1(10.8-15.3)	21.1(18.4-23.9)	8.2(6.4-10.0)
	65+ years	19.1	25.7(22.3-29.1)	4.5(3.0-6.1)	15.9(13.0-18.8)	9.6(7.3-11.8)	30.7(26.9-34.5)	3.8(2.4-5.2)	5.3(3.5-7.0)	5.7(3.9-7.6)	11.1(8.6-13.6)	4.0(2.5-5.5)
Gender (3843)	Men	46.9	11.7(9.8-13.5)**	3.5(2.5-4.4)**	8.4(6.8-10.0)*	6.4(4.9-7.9)	25.8(23.1-28.6)*	7.8(6.0-9.7)*	6.8(5.1-8.5)*	10.5(8.5-12.4)**	22.2(19.3-25.0)	5.6(4.3-6.9)***
	Women	53.1	15.4(13.8-17.1)	6.0(4.9-7.1)	10.9(9.5-12.3)	7.9(6.7-9.1)	29.8(27.6-32.0)	11.0(9.5-12.5)	9.1(7.7-10.4)	14.5(12.8-16.2)	25.4(23.4-27.5)	9.3(7.9-10.7)
Race/ethnicity (3806)	White, non-Hispanic	83.5	11.8(10.6-13.1)***	4.7(3.9-5.5)	10.2(9.0-11.4)	7.3(6.3-8.2)	27.4(25.6-29.3)*	9.1(7.9-10.3)	7.2(6.2-8.3)*	11.9(10.5-13.2)	23.1(21.3-24.9)*	7.2(6.2-8.3)*
	Hispanic	10.2	29.4(23.4-35.4)	5.2(2.6-7.8)	8.5(5.4-11.5)	6.3(3.7-8.9)	25.4(19.5-31.3)	10.1(6.5-13.7)	10.0(6.4-13.6)	16.0(11.4-20.5)	24.1(16.1-32.0)	7.2(4.3-10.2)
	Other	6.3	11.2(6.8-15.6)	5.7(2.1-9.3)	6.3(2.7-9.9)	7.9(1.0-14.9)	37.4(28.8-45.9)	13.6(6.1-21.0)	14.3(6.8-21.7)	17.5(9.9-25.0)	35.1(27.3-42.9)	12.9(7.3-18.5)
Income (3274)	<$25k	25.9	27.7(24.4-31.0)***	9.4(7.3-11.5)***	16.5(13.8-19.2)***	11.1(8.9-13.2)***	40.6(36.7-44.5)***	14.0(11.4-16.6)***	15.5(12.7-18.3)***	19.0(15.9-22.1)***	26.7(23.2-30.1)	12.2(9.7-14.7)***
	$25k-49,999	31.6	11.4(9.1-13.6)	4.1(2.8-5.4)	8.9(7.0-10.9)	7.8(5.5-10.0)	26.5(23.2-29.8)	10.6(8.0-13.2)	7.7(5.3-10.1)	13.9(11.2-16.6)	25.8(22.5-29.0)	7.3(5.5-9.2)
	$50k +	42.5	4.3(2.9-5.7)	3.0(1.9-4.1)	5.6(4.2-7.0)	4.6(3.3-5.9)	20.9(18.3-23.5)	6.4(4.7-8.1)	3.8(2.6-5.0)	8.4(6.6-10.2)	22.7(19.9-25.4)	5.4(3.9-6.8)
Employment (3829)	Unable to work	4.5	57.3(47.7-66.9)	34.3(25.8-42.9)	45.0(35.7-54.2)	45.5(35.8-55.3)	67.3(58.5-76.0)	35.7(26.1-45.3)	40.1(30.2-49.9)	37.5(27.7-47.4)	43.7(34.4-53.0)	34.5(25.5-43.6)
	Unemployed	5.5	24.8(17.6-32.1)	10.3(5.9-14.7)	14.4(9.4-19.3)	11.8(7.2-16.4)	39.4(31.7-47.2)	19.1(13.2-25.1)	18.2(12.3-24.0)	31.4(24.2-38.7)	28.9(22.0-35.7)	16.5(10.8-22.1)
	Retired	17.5	26.8(23.2-30.4)	4.8(3.1-6.4)	15.5(12.5-18.5)	9.1(6.8-11.3)	31.1(27.1-35.1)	4.7(3.0-6.3)	5.8(3.9-7.7)	7.9(5.6-10.3)	11.0(8.4-13.5)	4.8(3.0-6.6)
	Homemaker/Student	10.5	11.2(7.5-14.8)	4.2(1.9-6.4)	8.4(4.9-11.9)	2.7(1.1-4.3)	30.7(24.5-36.8)	10.3(6.6-13.9)	8.3(5.0-11.7)	11.6(8.0-15.3)	24.0(18.7-29.3)	6.9(3.9-9.9)
	Employed	62.0	6.3(5.1-7.4)***	2.4(1.7-3.1)***	5.5(4.4-6.5)***	4.3(3.4-5.2)***	22.9(20.8-24.9)***	8.0(6.6-9.4)***	5.4(4.3-6.5)***	10.7(9.2-12.2)***	25.6(23.3-28.0)***	5.9(4.8-7.0)***
Current smoker (3830)	Current smoker	22.4	14.1(11.5-16.6)	6.6(4.9-8.4)	12.2(9.7-14.7)	9.8(7.2-12.4)	32.9(28.8-37.0)	15.8(12.6-19.0)	14.2(11.1-17.3)	21.1(17.6-24.6)	32.7(28.7-36.7)	12.7(10.1-15.4)
	Not current smoker	77.6	13.5(12.0-14.9)	4.3(3.5-5.1)**	9.0(7.9-10.2)*	6.4(5.4-7.4)**	26.4(24.5-28.3)**	7.7(6.6-8.9)***	6.2(5.2-7.2)***	10.1(8.8-11.4)***	21.4(19.5-23.3)***	6.1(5.1-7.1)***
Chronic drinker (3798)	Chronic drinker	7.3	7.6(3.5-11.8)	3.5(1.4-5.7)	5.9(3.0-8.7)	9.8(3.8-15.8)	30.6(22.2-39.0)	14.8(7.5-22.0)	11.5(4.9-18.1)	21.3(13.7-28.9)	33.1(25.4-40.9)	8.1(4.6-11.7)
	Not chronic drinker	92.7	14.2(12.8-15.5)*	4.9(4.1-5.7)	10.1(8.9-11.2)*	7.0(6.1-7.9)	27.6(25.8-29.4)	9.0(7.9-10.1)	7.7(6.6-8.7)	11.8(10.6-13.0)**	23.2(21.5-25.0)**	7.6(6.6-8.6)
Activity (3841)	Leisure time activity	75.4	9.4(8.1-10.7)***	3.1(2.5-3.8)***	7.0(5.9-8.1)***	5.0(4.0-6.0)***	23.6(21.7-25.6)***	8.0(6.7-9.2)***	6.3(5.1-7.4)***	10.5(9.1-11.8)***	22.5(20.5-24.5)**	6.5(5.5-7.6)***
	No leisure time activity	24.6	26.7(23.6-29.7)	10.0(7.9-12.1)	18.2(15.5-21.0)	13.9(11.5-16.3)	41.4(37.5-45.2)	14.3(11.7-17.0)	13.4(10.9-16.0)	19.4(16.4-22.4)	28.4(25.0-31.8)	10.9(8.6-13.3)
Asthma (3824)	Asthma	8.9	24.5(19.5-29.5)	12.6(8.7-16.5)	22.9(17.7-28.1)	15.8(11.4-20.2)	43.6(37.3-49.8)	16.2(11.9-20.5)	14.8(10.5-19.2)	22.8(17.6-28.0)	33.6(27.8-39.4)	18.5(13.7-23.3)
	No asthma	91.1	12.5(11.3-13.8)***	4.1(3.4-4.8)***	8.5(7.4-9.5)***	6.4(5.4-7.3)***	26.5(24.7-28.3)***	8.8(7.6-10.0)***	7.4(6.3-8.5)***	11.7(10.4-13.0)***	23.0(21.2-24.9)***	6.6(5.6-7.5)***
Diabetes (3835)	Diabetes	5.6	40.2(33.2-47.2)***	12.0(7.3-16.8)***	20.2(14.5-25.9)***	19.3(13.5-25.1)***	43.2(35.8-50.7)***	14.3(9.0-19.6)*	15.0(9.6-20.5)***	18.4(12.4-24.4)*	19.2(13.4-25.0)	13.2(8.0-18.4)**
	No diabetes	94.4	12.0(10.8-13.2)	4.4(3.7-5.1)	9.1(8.0-10.2)	6.4(5.5-7.3)	27.0(25.2-28.8)	9.2(8.1-10.4)	7.6(6.5-8.7)	12.3(11.0-13.6)	24.2(22.4-26.0)	7.3(6.3-8.3)
Obesity (3605)	Obese (BMI>30)	18.5	23.0(19.3-26.7)	8.5(6.2-10.9)	14.2(11.2-17.2)	13.0(10.2-15.9)	36.5(32.2-40.9)	11.8(9.1-14.6)	11.6(9.0-14.3)	16.2(13.1-19.3)	27.2(23.2-31.2)	10.9(8.2-13.6)
	Not obese	81.5	11.4(10.1-12.7)***	4.1(3.3-4.9)***	8.7(7.6-9.9)***	5.9(4.9-7.0)***	26.2(24.2-28.1)***	8.9(7.5-10.2)*	7.1(5.8-8.3)***	11.7(10.3-13.2)**	22.9(21.0-24.7)*	6.7(5.6-7.7)**
Disability (3627)	Have disability	14.6	41.1(36.5-45.7)***	20.7(17.1-24.4)***	35.5(31.0-40.1)***	30.6(26.4-34.9)***	55.9(51.1-60.7)***	16.8(13.4-20.2)***	17.6(14.1-21.0)***	23.8(19.8-27.8)***	38.2(33.6-42.8)***	18.1(14.4-21.8)***
	No disability	85.4	8.2(7.1-9.3)	1.4(0.9-1.8)	5.1(4.3-6.0)	2.9(2.1-3.7)	21.9(20.1-23.7)	7.3(6.1-8.5)	5.5(4.4-6.5)	9.8(8.5-11.1)	21.0(19.1-22.8)	4.9(4.1-5.8)

[†]: Data are reported as weighted percents, and 95% confidence intervals (CIs) are reported in parentheses.
[‡]: Criteria is ≥ 14 days/month, see methods for complete variable description.
***: Statistically significant difference in variable proportions across groups, ***p<0.001; **p<0.01; *p<0.05.**
