# Supplementary material for: Associations between health-related quality of life and demographics and health risks. Results from Rhode Island's 2002 behavioral risk factor survey
Source: Health Qual Life Outcomes. 2006 Mar 3;4:14. doi: 10.1186/1477-7525-4-14 (PMC1431510; doi:10.1186/1477-7525-4-14)
Supplement: Additional File 4 — Table 2. Demographic characteristics and risk factors regressed on HRQOL indicators (Crude Odds Ratios) †, Rhode Island Adults, 2002 [file 1477-7525-4-14-S4.pdf]

Table 2. Demographic characteristics and risk factors regressed on HRQOL indicators (Crude Odds Ratios)† , Rhode Island Adults, 2002

Demographic characteristics & risk factors		Poor/fair general health	Activity limitation‡	Physically unhealthy‡	Pain related activity limitation‡	Lack of energy‡	Mentally unhealthy‡	Sad/blue/ depressed‡	Worried/tense /anxious‡	Lack of rest / sleep‡	Major depressive episode
Age group	18-44 years	Reference	Reference	Reference	Reference	Reference	Reference	Reference	Reference	Reference	Reference
	45-64 years	2.4(1.7-3.2)***	1.6(1.0-2.5)*	1.4(1.0-2.0)*	1.9(1.2-2.8)**	0.9(0.7-1.1)	0.8(0.6-1.2)	0.8(0.5-1.1)	0.8(0.6-1.1)	0.6(0.5-0.8)***	1.0(0.7-1.4)
	65+ years	4.9(3.5-6.7)***	1.6(1.0-2.6)	2.9(2.1-4.1)***	2.1(1.4-3.3)***	1.2(0.9-1.5)	0.3(0.2-0.5)***	0.6(0.3-1.0)*	0.3(0.2-0.5)***	0.3(0.2-0.4)***	0.5(0.3-0.8)**
Gender	Men	Reference	Reference	Reference	Reference	Reference	Reference	Reference	Reference	Reference	Reference
	Women	1.3(1.0-1.7)*	1.6(1.1-2.4)*	1.3(1.0-1.8)*	1.1(0.8-1.6)	1.2(1.0-1.4)	1.3(0.9-1.8)	1.3(0.9-1.9)	1.4(1.1-1.9)*	1.2(1.0-1.5)	1.6(1.1-2.3)**
Race/ethnicity	White, non-Hispanic	Reference	Reference	Reference	Reference	Reference	Reference	Reference	Reference	Reference	Reference
	Hispanic	3.3(2.3-4.7)***	0.7(0.4-1.4)	0.6(0.4-1.1)	0.8(0.4-1.3)	0.7(0.5-1.1)	0.7(0.4-1.3)	0.9(0.5-1.6)	1.1(0.7-1.8)	0.8(0.5-1.2)	0.8(0.4-1.5)
	Other	1.0(0.6-1.8)	0.9(0.3-2.2)	0.5(0.3-1.1)	1.1(0.3-3.6)	1.5(0.9-2.3)	1.7(0.8-3.6)	2.1(1.0-4.5)	1.4(0.7-2.8)	1.7(1.2-2.6)**	1.7(0.9-3.2)
Income	<$25k	9.1(6.2-13.1)***	2.7(1.6-4.3)***	3.3(2.3-4.6)***	2.2(1.5-3.3)***	2.3(1.8-3.0)***	1.9(1.3-2.8)**	3.9(2.6-6.0)***	2.3(1.6-3.2)***	1.2(0.9-1.5)	2.0(1.4-3.1)***
	$25k-49,999	3.2(2.1-4.8)***	1.5(0.9-2.6)	1.8(1.2-2.6)**	1.8(1.1-2.7)*	1.4(1.1-1.8)**	1.7(1.1-2.6)*	2.0(1.2-3.3)**	1.7(1.2-2.5)**	1.2(0.9-1.5)	1.3(0.9-2.0)
	$50k +	Reference	Reference	Reference	Reference	Reference	Reference	Reference	Reference	Reference	Reference
Employment	Unable to work	21.2(11.7-38.3)***	19.5(10.4-36.6)***	12.4(7.0-22.0)***	19.3(10.4-35.5)***	6.2(3.6-10.7)***	5.0(2.5-10.2)***	9.2(4.7-18.3)***	3.5(1.7-7.1)***	1.8(1.0-3.0)*	6.9(3.7-12.9)***
	Unemployed	5.9(3.7-9.3)***	4.7(2.4-9.2)***	4.0(2.4-6.6)***	3.7(2.1-6.4)***	2.3(1.5-3.4)***	2.7(1.7-4.5)***	3.7(2.2-6.4)***	3.7(2.4-5.6)***	1.2(0.8-1.9)	2.8(1.7-4.9)***
	Retired	5.3(3.9-7.2)***	2.7(1.6-4.4)***	3.5(2.5-4.9)***	2.2(1.5-3.3)***	1.5(1.1-1.9)**	0.5(0.3-0.8)**	0.9(0.6-1.6)	0.6(0.4-1.0)*	0.4(0.3-0.5)***	0.8(0.4-1.3)
	Homemaker/Student	1.8(1.1-3.0)*	1.5(0.7-3.2)	1.4(0.8-2.4)	0.8(0.4-1.7)	1.3(0.9-1.9)	1.2(0.7-2.1)	2.2(1.2-3.8)**	1.2(0.8-1.9)	1.1(0.7-1.6)	1.3(0.7-2.5)
	Employed	Reference	Reference	Reference	Reference	Reference	Reference	Reference	Reference	Reference	Reference
Current smoker	Current smoker	1.0(0.8-1.3)	1.3(0.8-1.9)	1.3(0.9-1.7)	1.4(0.9-2.1)	1.4(1.1-1.7)*	2.1(1.4-2.9)***	2.4(1.6-3.5)***	2.1(1.5-2.8)***	1.7(1.3-2.1)***	1.8(1.3-2.6)**
	Not current smoker	Reference	Reference	Reference	Reference	Reference	Reference	Reference	Reference	Reference	Reference
Chronic drinker	Chronic drinker	0.4(0.2-0.7)**	0.8(0.4-1.5)	0.6(0.4-1.1)	1.6(0.7-3.4)	1.4(0.9-2.2)	2.2(1.2-4.1)*	1.9(0.9-3.9)	1.9(1.1-3.3)*	1.7(1.1-2.5)**	1.2(0.7-2.1)
	Not chronic drinker	Reference	Reference	Reference	Reference	Reference	Reference	Reference	Reference	Reference	Reference
Activity	Leisure time activity	Reference	Reference	Reference	Reference	Reference	Reference	Reference	Reference	Reference	Reference
	No leisure time activity	4.0(3.1-5.1)***	4.0(2.7-5.9)***	3.5(2.6-4.6)***	3.2(2.3-4.5)***	2.3(1.8-2.9)***	2.0(1.4-2.8)***	2.4(1.7-3.5)***	2.2(1.6-2.9)***	1.6(1.3-2.0)***	1.8(1.2-2.5)**
Asthma	Asthma	2.2(1.5-3.2)***	3.0(1.8-5.1)***	2.7(1.9-4.0)***	2.9(1.9-4.4)***	2.0(1.5-2.8)***	1.7(1.0-2.6)*	1.9(1.2-3.2)**	2.1(1.5-3.2)***	1.6(1.1-2.2)**	3.3(2.2-5.1)***
	No asthma	Reference	Reference	Reference	Reference	Reference	Reference	Reference	Reference	Reference	Reference
Diabetes	Diabetes	4.1(2.8-6.0)***	2.4(1.3-4.4)**	2.4(1.5-3.7)***	2.8(1.7-4.6)***	1.8(1.3-2.6)**	0.7(0.4-1.5)	1.3(0.7-2.3)	1.0(0.5-1.7)	0.4(0.3-0.7)***	1.2(0.6-2.2)
	No diabetes	Reference	Reference	Reference	Reference	Reference	Reference	Reference	Reference	Reference	Reference
Obesity	Obese (BMI>30)	2.1(1.6-2.8)***	2.1(1.3-3.1)***	1.8(1.3-2.4)***	2.2(1.6-3.2)***	1.6(1.3-2.0)***	1.1(0.8-1.6)	1.4(0.9-2.0)	1.3(0.9-1.7)	1.2(0.9-1.5)	1.4(1.0-2.1)
	Not obese	Reference	Reference	Reference	Reference	Reference	Reference	Reference	Reference	Reference	Reference
Disability	Have disability	7.9(6.0-10.3)***	19.3(12.5-29.7)***	10.1(7.5-13.7)***	13.9(9.5-20.4)***	4.4(3.5-5.7)***	2.4(1.7-3.4)***	3.7(2.6-5.3)***	2.8(2.1-3.8)***	2.4(1.8-3.0)***	4.0(2.8-5.8)***
	No disability	Reference	Reference	Reference	Reference	Reference	Reference	Reference	Reference	Reference	Reference

†: Data are reported as crude odds ratios (CORs), 95% confidence intervals (CIs) are reported in parentheses.
‡: Criteria is ≥ 14 days/month, see methods for complete variable description.
***: Statistically significant, ***p<0.001; **p<0.01; *p<0.05.**
